# New Books

**Published:** 2010-04

**Climate Refugees**

Collectif Argos

Cambridge, MA:MIT Press, 2010. 349 pp. ISBN: 978-0-262-51439-2, $29.95

**Ecosystem Geography: From Ecoregions to Sites, 2nd ed.**

Robert G. Bailey

New York:Springer, 2010. 243 pp. ISBN: 978-1-4419-0391-4, $49.95

**Evaluation of Certain Veterinary Drug Residues in Food**

World Health Organization

Geneva:WHO Press, 2009. 141 pp. ISBN: 978-9-2412-0954-0, $25

**Greening through IT: Information Technology for Environmental Sustainability**

Bill Tomlinson

Cambridge, MA:MIT Press, 2010. 216 pp. ISBN: 978-0-262-01393-2, $24.95

**Hayes’ Handbook of Pesticide Toxicology, 3rd ed.**

Robert Krieger

St. Louis, MO:Elsevier, 2009. 2,000 pp. ISBN: 978-0-12-374367-1, $595

**Learning Science in Informal Environments: People, Places, and Pursuits**

Philip Bell, Bruce Lewenstein, Andrew W. Shouse, Michael A. Feder, eds.

Washington, DC:National Academies Press, 2009. 352 pp. ISBN: 978-0-309-11955-9, $44.96

**Living Through the End of Nature: The Future of American Environmentalism**

Paul Wapner

Cambridge, MA:MIT Press, 2010. 184 pp. ISBN: 978-0-262-01415-1, $21.95

**Losing Our Cool: Uncomfortable Truths About Our Air-Conditioned World**

Stan Cox

New York:New Press, 2010. 272 pp. ISBN: 978-1-59558-489-2, $24.95

**Marine Ecology: Concepts and Applications**

Martin R. Speight, P. A. Henderson

Hoboken, NJ:John Wiley & Sons, 2010. 256 pp. ISBN: 978-1-4051-2699-1, $150

**Principles for Modeling Dose-Response for the Risk Assessment of Chemicals**

World Health Organization

Geneva:WHO Press, 2009. 124 pp. ISBN: 978-9-2415-7239-2, $35

**Sea Ice, 2nd ed.**

David N. Thomas, Gerhard S. Dieckmann, eds.

Hoboken, NJ:John Wiley & Sons, 2010. 640 pp. ISBN: 978-1-4051-8580-6, $152.99

**Southeast Asia: The Long Road Ahead**

Chong Yah Lim

Hackensack, NJ: World Scientific, 2009, 504 pp. ISBN: 978-981-4280-80-8, $72.00

**Treading Softly: Paths to Ecological Order**

Thomas Princen

Cambridge, MA:MIT Press, 2010. 224 pp. ISBN: 978-0-262-01417-5, $22.95

**WHO Guidelines for Indoor Air Quality: Dampness and Mould**

WHO Regional Office for Europe

Geneva:WHO Press, 2009. 247 pp. ISBN: 978-9-2890-4168-3, $50

**Xenobiotics in the Urban Water Cycle: Mass Flows, Environmental Processes, Mitigation and Treatment Strategies**

Despo Fatta-Kassinos, Kai Bester, Klaus Kümmerer, eds.

New York:Springer, 2010. 480 pp. ISBN: 978-90-481-3508-0, $199

Arnold Greenwell/EHP

